# The impact of restrictions due to covid-19 on mental health of adolescents including the incidence of the social anxiety disorder and progression of already existing social anxiety symptoms

**DOI:** 10.1192/j.eurpsy.2023.1494

**Published:** 2023-07-19

**Authors:** A. Pentjuša

**Affiliations:** University of Latvia, Riga, Latvia

## Abstract

**Introduction:**

The Covid-19 pandemic has brought many changes to everyday life of adolescents. The purpose of this study is to evaluate the impact of restrictions due to Covid-19 pandemic on the mental health of adolescents: changes in everyday life that can potentially have an impact on their mental health, the prevalence and worsening of social anxiety symptoms and consequent depression symptoms

**Objectives:**

To evaluate the impact of restrictions due to Covid-19 pandemic on the mental health of adolescents: changes in everyday life that can potentially have an impact on their mental health, the prevalence and worsening of social anxiety symptoms and consequent depression symptoms

**Methods:**

Anonymised questionnaires were used to collect the data. They were given to the patients staying in the department of psychiatry of Children’s Clinical University Hospital in Riga, Jugla as well as to some adolescents visiting child psychiatrist in outpatient settings from January till the end of April, 2022. The following personal data were collected - age, gender, family status - as well as information about different factors affecting the mental health of adolescents during the pandemic: how often they spent time with friends, whether or not they have lost any friends, distance learning, seeking help from mental health professionals, quality of sleep and a chance to receive emotional support. Patients also filled Liebowitz social anxiety scale and PHQ-9: modified for adolescents’ depression scale.

**Results:**

Restrictions due to pandemic mostly affect the participants negatively, promoting the worsening of social anxiety symptoms in 42% of the respondents with positive results of the Liebowitz scale. Statistically significant connection between social anxiety and depression symptoms was found. During the pandemic most of the patients were more often seeking professional help. Patients with worsening social anxiety symptoms were found to have statistically significant connection to losing friends during the pandemic. Most of the recipients with already diagnosed social anxiety were given this diagnosis during the pandemic (67% of the cases).

**Conclusions:**

The restrictions due to Covid – 19 pandemics negatively affect adolescents including those with social anxiety, promoting the worsening of symptoms as well as prevalence of depression symptoms in these individuals. The results suggest that coping strategies must be implemented in order to decrease the consequences of the pandemic on adolescents.

**Disclosure of Interest:**

None Declared

